# Old European Silkworm Breeds Reared in Early-20th-Century Bulgaria and Their Potential Use in Modern Sericulture

**DOI:** 10.3390/insects16121246

**Published:** 2025-12-09

**Authors:** Panomir Tzenov, Dimitar Grekov, Velislav Iliev

**Affiliations:** 1 Agricultural Academy, Scientific Center on Sericulture, 3000 Vratsa, Bulgaria; 2 Agronomy Faculty, Agricultural University, 3000 Plovdiv, Bulgaria; 3 Plant Protection and Agroecology Faculty, Agricultural University, 3000 Plovdiv, Bulgaria

**Keywords:** sericulture, silkworm, *Bombyx mori* L., breeds, old, breeding, improvement

## Abstract

Since local egg production using Pasteur’s method began in 1895, two local silkworm breeds have mostly been reared in Bulgaria, namely, Yellow local and White Baghdad. The Yellow local breed is said to have originated and been reared from local breeds spread in northern and southwestern Bulgaria in the 16th–19th centuries, while White Baghdad originated from Asia Minor and was reared in southeastern Bulgaria. The Yellow local breed is characterized by yellow cocoons with an elongated shape with slight constriction. The white Baghdad breed consists of three types, namely the Edirne, Improved Bulgarian, and Bulgarian types. The cocoon color varies from snow white to light green, but the prevailing color is white. The cocoon shape is elongated, with constriction. The old silkworm breeds reared in 1920s–1930s Bulgaria were generally characterized by a non-uniformity of larval color and markings, as well as of cocoon color and shape, with comparatively high cocoon weights but lower silk shell percentages and filament lengths; they were also comparatively tolerant to NPV disease. Due to the long maintenance of these breeds in Bulgaria, they have become well adapted to the local food and climatic conditions. Presently, the Yellow local strain displays high hatchability and survivability, shorter fifth-instar duration, and a comparatively good reproduction capacity. On the other hand, it manifests lower values of main productive characteristics, such as cocoon weight, silk shell weight and percentage, silk filament length and weight, reelability, and raw silk percentage as compared with commercial Bulgarian white-cocoon breeds. Therefore, in order for this breed to be commercialized today, it needs to be improved by way of genetics and breeding. This paper includes a review of the literature on the inheritance of main qualitative characteristics in silkworms, as well as recommendations for future studies on improving old silkworm breeds.

## Simple Summary

This paper focuses on the history of Bulgarian sericulture and the characteristics of silkworm breeds used towards the end of the 19th and the first half of the 20th century in Bulgaria. Old silkworm breeds reared in Bulgaria during the 1920s and 1930s were generally characterized by a non-uniformity of larval color and markings, as well as of cocoon color and shape, with comparatively high cocoon weights but lower silk shell percentages and filament lengths; in addition, they were comparatively tolerant to NPV disease. Data on the performance of the old Bulgarian silkworm breed Yellow local in 2023–2024 are presented and discussed. The breed manifested lower values of main productive characteristics, such as cocoon weight, silk shell weight and percentage, silk filament length and weight, reelability, and raw silk percentage, as compared with modern commercial white-cocoon breeds from Bulgaria. Commercialization of the Yellow local breed would require improvement via genetics and breeding. This paper presents a literature review on the inheritance of the main qualitative characteristics of silkworm, including recommendations for future research work on improving old silkworm breeds.

## 1. Introduction

A very important element in connecting European identity with its historical past is the preservation and even reintroduction of traditions and tangible elements and engaging contemporary Europeans with their historical past. In the case of sericulture, one such tangible element are the indigenous (European) breeds of silkworm, which offer European cocoon producers a link to their traditions and identity. A number of countries have made efforts aimed at the conservation of agrobiodiversity in an attempt to prevent the loss of genetic diversity of plants and animals, including the silkworm [1,2,3,4,5,6,7,8,9,10]. By providing the opportunity to rear indigenous silkworm breeds, modern practices could be linked to local traditions. It is clear that this would be a slow and difficult process that would require complex actions, but it is the only way to maintain the traditions, local agrobiodiversity, and local identity of the European regions that are closely linked to silk production and silkworm rearing. Most of the old local European breeds of silkworms, such as Yellow local in Bulgaria, Brianza in Italy, Var in France, Sierra Morena in Spain, and Kakhetian Green and Kutaisi Orange in Georgia, etc., are characterized by colored cocoons [11,12,13,14,15,16,17,18,19,20,21,22,23]. At the same time, after the 1950s, almost all countries with developed sericulture switched to the mass breeding of white-cocoon hybrids. In addition to the fact that this led to a decrease in local biodiversity, the use of imported silkworm hybrids proved unsuccessful in preventing the decline in sericulture in Europe, as observed in the second half of the 20th century. Although they were once considered a way to improve silk production in Europe, due to these imported white-cocoon hybrid silkworms, local and other colored-cocoon breeds were, in practice, no longer the targeted selection for vitality and productivity but were only maintained as a gene pool. The lack of targeted breeding and improvement work has led to relatively low values of the main quantitative traits shaping the productivity and quality of silk in breeds with colored cocoons, compared to the white-cocoon breeds used for industrial hybridization. In order to improve the main quantitative traits in breeds with colored cocoons, it is necessary to cross them with highly productive white-cocoon breeds and undertake subsequent selection in order to consolidate valuable economic traits in the offspring while preserving the qualitative traits typical of these breeds, such as the color of the larvae and the color and shape of the cocoon. In this respect, it is important to study the qualitative and quantitative traits of old European breeds from Bulgaria in the first half of the 20th century, when those breeds were widely used as the basis of the sericultural industry.

## 2. The Bulgarian Sericulture Industry at the End of the 19th Century and the First Half of the 20th Century: Local Silkworm Breeds and Egg Production

This section is dedicated to the historical development of the sericultural industry in Bulgaria, as well as to the local and introduced breeds of silkworms bred in the 19th and first half of the 20th centuries. In fact, sericulture in Bulgaria, like in many other countries, has gone through stages of both rapid development and decline. We synthesize Bulgarian and non-Bulgarian sources to trace how disease ecology, trade policy, and labor migration jointly structured sericulture capacity.

Regarding the sources used for this study, we could say that the overwhelming percentage of them are reliable, such as published scientific and popular articles, books, annual scientific reports and administrative reports, and studies based on reliable historical documents.

Sericulture reached the Balkan peninsula in the 7th–8th century AD [24,25,26,27,28,29]. It is believed that sericulture was adopted in Bulgaria during the first Bulgarian empire, towards the end of the 10th century AD [17,30] (Table 1).

It has been reported that in the 13th and 14th centuries AD, in the second Bulgarian empire, there were some villages and single households whose main income was derived from sericulture and silk production [26,28,31,32,33]. Tudelski, a Spanish traveler from the 12th century AD, wrote that sericulture in the Balkan peninsula was so well developed that the cities were full of silk [28,34,35].

During the Russian–Turkish war for the liberation of Bulgaria of 1877–1878, many mulberry trees were cut [22]. After this war, there was a mass migration of the local Turkish population to Turkey, and the labor force decreased. The fertile lands that were left behind were used for cereal production by local Bulgarians, and so sericulture was almost forgotten [29,36,37]. By 1880, due to the above reasons—and also due to the spread of pebrine disease—sericulture had almost disappeared from the newly liberated Bulgaria [38,39].

Pebrine is a silkworm disease caused by the parasite *Nosema bombycis*, and Louis Pasteur’s method to control it involved microscopic examination and selection. After the moths laid their eggs, Pasteur crushed the adult moths and examined the resulting pulp under a microscope. If parasitic spores were found, the eggs from that batch were destroyed; otherwise, the healthy eggs were kept for future breeding. This method allowed for the selection of non-infected breeding stock, which was crucial for restarting the French silk industry devastated by the disease.

After 1890, there was an economic crisis in Bulgaria, leading to widespread poverty. Since sericulture was a good source of income, cocoon production increased very rapidly. For example, in 1896, about 3 million mulberry saplings were produced and planted in Bulgaria [18,23]. In addition, many mulberry saplings were imported from Turkey [23,27]. In 1892, the first silk-reeling factory, with 1000 ends, was established in southern Bulgaria [14,24]. It was equipped with multi-end Batalia system reeling machines from Italy, which were comparatively modern for the time [14].

The first statistical data on fresh cocoon production in Bulgaria are from 1886, when 291 tons of fresh cocoons were produced [30] (Table 1).

In 1896, the Sericulture Experiment Station was established in Vratsa [15,17,23,27,40].

In the 12 years from 1890 to 1902, fresh cocoon production in Bulgaria increased by about 10 times and reached about 1000 tons annually [37]. This period of flourishing for sericulture continued until the First Balkan War, the Inter-Allied Wars, and the First World War. These wars led to a sharp decline in cocoon production. However, soon after the wars, due to increased market demand for silk, cocoon prices increased as well [23,29]. In 1929, fresh cocoon production in Bulgaria amounted to 2400 tons [23]. An analysis of the Bulgarian export trade in 1928 showed that dry cocoons occupied the fourth place by value among the Bulgarian goods exported [17,24]. During the period of 1925–1929, the number of sericulture households was 70,000–80,000 [17].

After 1929, silk prices in the international market sharply and suddenly decreased, which was reflected in the prices of cocoons: at the time, most cocoons were exported to Milan and Marseille [23]. As a result, annual fresh cocoon production dropped from 2300 tons in 1930 to only 1100 tons in 1931 [14,23].

Until 1930, Bulgarian traders were satisfied with exporting dry cocoons due to their good prices. With the decrease in cocoon prices and difficulty finding buyers, some traders tried to process the cocoons within Bulgaria; as a result, in just a few years, dozens of silk-reeling mills were established and started to reel all the cocoons produced in Bulgaria [29]. At the same time, some silk-weaving mills were also established. In order to stimulate the development of the local silk industry, the government established protective duties for imported silk goods [23,33]. Silk-weaving mills were opened in the cities of Ruse, Vratsa, Plovdiv, Sofia, and Karlovo. For a comparatively short period of time, in the 1930s, a considerable silk industry was established in Bulgaria. This industry included 1500 silk-reeling mills, with a processing capacity of 3000 t fresh cocoons annually; 480 mechanical silk-weaving looms, with an annual capacity of about 1,400,000 m silk fabric; silk-twisting machines with 10,000 spindles and an annual capacity of 60 tons of twisted silk; and 15 silk sock-knitting machines, with an annual capacity of 100,000 pairs of socks [14,15,23,29,41,42,43].

**Table 1:** More important periods of Bulgarian sericulture development.

Period	Event	Sources	Reliability Notes Concerning the Sources of Information
7th–8th century AD	Sericulture was adopted in the Balkan peninsula.	[24,25,26,27,28,29]	Not very reliable
10th century AD	Adoption of sericulture in Bulgaria.	[17,30]	Not very reliable
1840	Boom of cocoon and home silk production.	[30,37]	Reliable
1865	Canceling the tax on silk by the Turkish government in order to stimulate sericulture development.	[30,32,33,38]	Reliable
1870–1880	Big decline in cocoon and silk production due to pebrine disease spreading and Russian–Turkish war for Bulgaria liberation.	[17,24,30,36]	Reliable
1886	291 tons of fresh cocoons were produced.	[29,34,35,44]	Reliable
1892	The first silk-reeling factory, having 1000 ends, was established in southern Bulgaria. It was equipped by Italian multi-end reeling machines, Batalia system, comparatively modern for that time.	[23,24,30,39]	Reliable
1895	The first silkworm eggs by Pasteur’s method were produced.	[23,27,29]	Reliable
1895–1960	Two indigenous silkworm breeds, namely, Yellow local and White Baghdad were cultivated.	[16,27,40,45]	Reliable
1896	The Sericulture Experiment Station in Vratsa was opened.	[23,25,29,30,33,37]	Reliable
1896	A law for silk industry development was passed. By this law, cocoon markets were created.	[23,24,29,30,33,37]	Reliable
1902	1000 tons of fresh cocoons were produced.	[24,30]	Reliable
1926	The largest silkworm egg production in the amount of 214,000 boxes (12 g) was reached.	[23,27,44,46]	Reliable
1927	The first phenological observations on mulberry were conducted at SES-Vratsa.	[22,46]	Reliable
1928	The dry cocoons occupied 4th place by value among the Bulgarian goods exported.	[24]	Reliable
1929	2400 tons of fresh cocoons were produced.	[22]	Reliable
1931	The fresh cocoon production dropped to 1100 tons annually.	[23,29]	Reliable
1932	The government made a decision that the Bulgarian Agricultural and Cooperative Bank were to provide conditions for cocoon production rehabilitation and increase.	[23,41,42,47]	Reliable
1944	The parliament voted on a special law about sericulture, aiming at the implementation of measures for the enlargement of mulberry plantations and cocoon production.	[27,29]	Reliable
1947	The “Cooperative of Bulgarian silkworm egg producers” was created.	[27,40,45]	Reliable
1948	The state enterprise “Textile fibers”, having branches in all the cocoon production regions, was established.	[27,40,45]	Reliable
1950	The entire egg production industry was taken by the Textile fibers state enterprise and centralized in 4 big factories.	[29,45]	Reliable
1953	The peak of cocoon production was reached, when 3019 tons of fresh cocoons were produced by a national country’s population of about 7 million inhabitants.	[27,29,44,46]	Reliable
1989	1500 tons of fresh cocoons were produced.	[48]	Reliable
1995	150 tons of fresh cocoons were produced.	[48]	Reliable
1997	Privatization of the companies dealing with cocoon and silk processing.	[49]	Reliable
2000–2002	TCP project “Rehabilitation of sericulture in Bulgaria”, financed by FAO.	[49]	Reliable
2018	Transforming SES-Vratsa into the Scientific Center on Sericulture under the Agricultural Academy.	[48]	Reliable
2023–2025	SCS-Vratsa preserves 204 mulberry varieties and 100 silkworm breeds at its germplasm.	[48,49]	Reliable

The first silkworm eggs, produced using Pasteur’s method, were imported to Bulgaria in 1883 [15,33]. Then, local egg production using Pasteur’s method started in 1895, when 2100 boxes (12 g) were produced [37].

In the past, silkworm eggs were measured and sold by the ounce. After the mid-20th century, silkworm eggs have been sized and sold in boxes, with a standard box containing 20,000 viable eggs or weighing about 12 g.

In parallel with locally produced eggs, until 1930, some silkworm eggs were also imported from France, Italy, and Turkey [23]. The greatest volume of local silkworm egg production was reached in 1926, when 214,000 boxes (12 g) were produced [27] (Table 1). After 1931, local egg production stabilized at around 125,000 boxes (12 g) annually for a long period of time [44]. A part of the locally produced silkworm eggs was exported to Iran, Romania, Poland, Yugoslavia, Czechoslovakia, the USA, and India, etc. [45], which evidences the high quality of the eggs produced. Starting in 1896, the Sericulture Experiment Station in Vratsa organized many training courses for silkworm egg producers [14,23,47]. At the same time, some egg producers graduated from training courses held at sericulture stations in Bursa, Montpellier, Padua, Ascoli, Piceno, and Tbilisi, etc. [40,45]. Until 1947, local egg production was concentrated in the hands of 60–85 private producers, working mainly in the regions of Vratsa, Teteven, Bjala Slatina, Botevgrad, Harmanli, Svilengrad, Ivailovgrad, Haskovo, Plovdiv, and Asenovgrad [27,40,50]. In 1947, the “Cooperative of Bulgarian silkworm egg producers” was created, and in 1950, egg production was overtaken by the state enterprise of textile fibers [40,45]. Egg production was centralized in four factories, situated in Vratsa, Berkovitsa, Plovdiv, and Harmanli [44,46]. In 1996, the egg production factory in Vratsa and the Sericulture Experiment Station were moved to the Agricultural Academy, and the other three factories remained in Sirma JSC; after its privatization in 1997, the egg production factories were closed.

Since local egg production using Pasteur’s method began in 1895, two local silkworm breeds have mostly been reared in Bulgaria, namely, Yellow local and White Baghdad. The origin of the Yellow local breed is said to be from a local strain spread in northern and southwestern Bulgaria from the 16th to the 19th centuries [51]. White Baghdad originated from Asia Minor [13].

The Yellow local breed was reared in all of northern and southwestern Bulgaria. It was characterized by a yellow cocoon color, and the cocoons had an elongated shape with slight constriction [12,16].

The White Baghdad breed was reared in southeastern Bulgaria and consisted of three types, namely, the Edirne type, Improved Bulgarian type, and Bulgarian type [19]. The cocoon color of the White Baghdad breed varied from snow white to light green, but the prevailing color was white [20]. The cocoon shape was elongated with constriction [21]. The local silkworm breeds were grown mostly as pure breeds until the 1960s; after that, they were gradually overtaken by white-cocoon hybrids [44,52]. The Yellow local breed is still preserved at the Scientific Center on Sericulture, Vratsa. A breed called “White Bulgarian”, which is supposed to originate from one of the White Baghdad types, is also maintained at the Center [48,49].

## 3. Characteristics of the Silkworm Breeds Reared and Tested in Bulgaria During the First Half of the 20th Century

In 1931–1932, Dushev [16] studied the larval color and markings of the Italian silkworm breeds Perudjia, Majela, Abruco, Askoli, Grand Saso, Umbrija, Bione, and Brianca, the French breeds Var and Yellow Alpin, and the local Bulgarian silkworm breeds Yellow local and White Baghdad at the Sericulture Experiment Station in Vratsa. Based on the description of the different breeds and types, the color of their skin varied from white, zebra, gray–brown, dark brown, gray–reddish brown, and chestnut to velvet. It should be noted, however, that the predominant color among all breeds and types, accounting for 66.40%, was white. It was followed by zebra (20.78%), gray–brown (8.4%), dark chestnut (3.36%), chestnut (1.48%), gray-reddish (0.84%), and velvet (0.84%). By virtue of their masks and the crescents that occur on the second, fifth, and eighth vertebrae, the larvae were divided into three categories: stronger, medium, and weak color intensity. The larval color and markings observed in the local Bulgarian breeds are presented in Table 2.

Within the main larval marking groups, however, the larvae were classified as follows:

Yellow local breed: 1. White, no mask and no moons. 2. White, medium mask, weak moons. 3. White, strong mask, strong moons. 4. Zebras, medium mask, no moons. 5. Zebras, strong mask, strong moons. 6. Gray–brown, no moons, strong mask. 7. Gray–brown zebras, no moons, strong mask. 8. Gray–brown, zebras, with moons, and strong mask.

White Baghdad breed: 1. White, no mask and no moons. 2. White, weak mask, weak moons. 3. White, medium mask, weak moons. 4. White, stronger mask, weak moons. 5. White, strong mask, strong moons. 6. Zebras with pigment, no moons, and medium mask. 7. Zebras with a strong mask, without moons. 8. Zebras with a strong mask, weak moons, and with pigment. 9. Zebras with a strong mask, strong moons. 10. Gray–brown, light mask, around the false legs are light pink spots with weak moons. 11. Gray–brown with a darker mask, around the false legs are light pink spots with stronger moons. 12. Gray–brown, darker mask, around the legs are brick spots with weak moons. 13. Gray–brown, darker mask, around the legs are brick spots with strong moons. 14. Gray–brown, zebras, strong mask, strong moons. 15. Gray–brown, zebra-like, strong mask, strong moons, brown–red spots around the legs.

It is evident that the local silkworm breeds reared in Bulgaria at the time varied greatly in larval color and markings.

According to Dushev [12], in order to meet the country’s needs for yellow-cocoon silkworm breeds, in 1932, the Bulgarian Agricultural Bank delivered 7000 ounces of purebred yellow-cocoon silkworm eggs from Italy to be distributed to sericulturists in northern Bulgaria. Simultaneously, the bank also purchased eggs of the local yellow-cocoon breed produced by local egg producers; in 1932, these amounted to 3340 ounces. The silkworm eggs purchased by the bank, both foreign and local, were stored in the winter storage of the SES in Vratsa, which was tasked, with the help of the district agronomists, with organizing their distribution among sericulturists who reared the larvae of the yellow-cocoon breed. The station distributed the eggs according to a strictly defined plan, with Italian eggs being sent to some regions and those of local origin to others. In this way, it was possible to prevent the cocoons obtained from the two groups of breeds from mixing and to see the quality of the cocoons obtained from the Italian and local yellow-cocoon breeds on a larger scale. The following studies were carried out on the cocoons and the silk obtained from them: (1) observations on the color, shape, and structure of cocoons; (2) dimensions of cocoons; (3) raw silk renditta of cocoons; (4) length of silk thread per cocoon; (5) silk titer; and (6) elasticity and strength of the silk thread. (Table 3 and Table 4) In terms of cocoon color and shape, there was no marked difference between the cocoons of the Yellow Italian and the Yellow local breeds. However, a larger difference emerged in terms of the nature of cocoon grains of these two types: the cocoons of the Yellow Italian breed had a finer structure than those of the Yellow local breed.

In terms of size and yield, the cocoons of the Yellow Italian and Yellow local breeds did not differ much from each other. In terms of titer, the silk titer of the Yellow Italian breed (Grade I and Grade II cocoons) was higher than the Yellow local breed. In terms of elasticity and strength, Grade I silk of the Yellow Italian breed achieved higher values; however, the difference between it and the silk of the Yellow local breed was only slight. Considering the qualitative differences obtained in the study and the study methodology, the general conclusion was that, in terms of cocoons and silk, there were slight differences between the Yellow Italian and Yellow local breeds. Any superiority on the part of either the Yellow Italian breed or the local breed was more due to larval feeding than to the qualities of the breed itself.

As revealed by Dushev’s [19] studies on the local White Baghdad breed, the variety in color found among the breeds’ larvae indicated that the White Baghdad breed reared in Bulgaria was not a pure breed but a mixture of different breeds (White Bursen and White Edirne). The French White Baghdad breed, which was also studied, represents a much greater degree of uniformity in this respect than the Bulgarian White Baghdad breed, perhaps thanks to more systematic selection on the part of the egg producers. The local White Baghdad breed resisted grasseria (NPV) disease more than the Yellow breed, the Golden Chinese, and the crossbreeds; however, this breed had no immunity to the disease. Compared to the Yellow Italian and the Golden Chinese breeds, moths of the White Baghdad breed lived almost twice as long as the Italian breed and three times as long as the Chinese breed.

The cocoons of the Bulgarian White Baghdad breed did not have one established typical white color, as may have been the case for the White Chinese breed. The white color of the Bulgarian White Baghdad breed was broken down into 5–8 different types of colors, which varied from snow white to reseda. In the cocoons, the predominant white color was snow white with a milky tint; it was also found at the highest percentage among the cocoons. The percentage of the snow white color in Bulgarian Baghdad cocoons reached only 10%, while in French Baghdad cocoons, it was almost 50%.

As for the color of the White breeds from France, it should be noted that especially for the White breed, the cocoons were distinguished by its predominant snow white color. Almost all groups of this type had a markedly uniform color, which attracted attention.

The Yellow local breed had the following two main colors: pale pink with a yellow tint and pale pink with an orange tint. It was noted that in color, the cocoons of all groups showed significant homogeneity. All this indicated a comparative purity of the yellow-cocoon breed reared in Bulgaria.

By shape, the cocoons of the Bulgarian White Baghdad breed were divided into three categories: weakly attached, medium-, and strongly attached. Cocoons of the French White Baghdad breed only had two shapes: weakly attached and medium-attached. Strongly attached ones were absent. The highest percentage (45%) of the Bulgarian White Baghdad cocoons were medium-attached, followed by strongly attached (20%). The predominant structure of the Bulgarian White Baghdad breed was medium-grained. However, the coarse-grained structure prevailed over the fine-grained. The average cocoon volume of the Bulgarian White Baghdad breed was 10 cubic cm. Male cocoons of the Bulgarian White Baghdad breed had an average length of 4 cm, and female cocoons 4.44 cm. The length of the male and female cocoons of the French White Baghdad breed was smaller. The average width of male Bulgarian White Baghdad cocoons was 1.93 cm, and that of female cocoons was 2.09 cm. In terms of width, cocoons of the French White Baghdad breed were almost the same as the Bulgarian White Baghdad breed. A certain asymmetry was noticeable in both the male and female cocoons of the Bulgarian White Baghdad breed: one side (the right) of the cocoon was wider than the other (the left). In male cocoons, the difference in width between the right and left side was, on average, 0.095 cm, and in female cocoons, it was 0.10 cm. The average weight of male cocoons of the Bulgarian White Baghdad breed was 2.378 g, with a maximum weight of 3.210 g; the average weight of the silk shell was 0.390 g. The average weight of female cocoons ranged from 3.076 to 3.326 g, and the silk shells were 0.463 g in weight. The percentage of double cocoons in the Bulgarian White Baghdad breed ranged from 1.6 to 3; that of under-wrapped cocoons was from 0.6 to 1.37, and that of satin cocoons from 0.01 to 0.04. The average renditta from the local White Baghdad breed in industrial cocoon reeling was 3.3591 kg for males and 3.6088 kg for females. From the same group of cocoons, in single-cocoon reeling, the average renditta was 3.0015 kg for male cocoons and 3.2516 kg for females. The percentage of frizon-to-cocoons in the Bulgarian White Baghdad breed was 9.03. The average length of silk thread in male cocoons of the Bulgarian White Baghdad breed was 808.64 m, with a maximum value of 1153 m and a minimum value of 600 m; in female cocoons, the average length was 891 m, and the maximum length was 1500 m. The average length of thread from the Bulgarian White Baghdad breed exceeded that from the French White Baghdad breed by 62 m. Nowadays, in order to measure the silk productivity, instead of characterizing “renditta”, it is more popular to characterize “raw silk percentage”. In the modern uni-bivoltine silkworm breeds, this character usually varies from 35 to 42%. If re-calculate the renditta to raw silk percentage using the data available for the Bulgarian White Baghdad breed, it is only 29.77%, while for Yellow local it is 31.33% or too low.

It can be concluded that the old silkworm breeds reared in Bulgaria during the 1920s and 1930s were generally characterized by a non-uniformity of larval color and markings, as well as of cocoon color and shape; they had comparatively high cocoon weights but lower silk shell percentages and filament lengths, and they were comparatively tolerant to NPV disease. Due to the long maintenance of these breeds in Bulgaria, they have become well adapted to the local food and climatic conditions.

How do historical morphological and quantitative traits relate to today’s breeding goals like disease resistance, climate resilience, and filament quality? Obviously, the old silkworm breeds do not meet the modern breeding requirements concerning breed uniformity and silk productivity. On the other hand, they possess higher diseases resistance and good adaptivity to the local mulberry varieties and climate. In order for the old breeds to be used in the sericulture field today, they should be improved by the methods of modern genetics and breeding, at the same time, keeping to the maximum extent their qualitative characteristics, diseases resistance, and local adaptability.

## 4. Potential Uses of Some of the Old European Breeds Reared in Bulgaria in Modern Sericulture

### Present Status of Old European Breeds in Bulgaria

Until the 1960s, the Yellow local strain was grown mostly as a pure breed [29,45]; after that, it was gradually overtaken by white-cocoon hybrids. Presently, among of the two old silkworm breeds—White Baghdad and Yellow local—only the germplasm of Yellow local is maintained at the Scientific Center on Sericulture (SCS) in Vratsa.

The following characteristics were detected in 2023 and 2024 [48,49]: hatchability, total larval period and fifth-instar duration, pupation rate, fresh cocoon weight, silk shell weight, silk shell ratio, fresh cocoon yield by one box of silkworm eggs, silk filament length and weight, reelability, and raw silk percentage. The study focused on the Yellow local breed and the breed Super 1 (white cocoon, Japanese type), while as the control for comparison, the popular Bulgarian white-cocoon breed Hesa 2 (Chinese type), the parent of the widely adopted Bulgarian F1 hybrid Super 1 × Hesa 2, and the reciprocal cross was used. The data obtained were processed mathematically using the methods of variational statistics, namely, through analysis of variance, and are presented in Table 5 and Table 6.

It is evident from the data obtained that the Yellow local silkworm strain achieved high hatchability. The total larval period duration was not much different compared with the controls, but the fifth-instar duration was significantly shorter in the Yellow local strain. The pupation rate was higher than that of Hesa 2 and lower than that of Super 1. Fresh cocoon yield by one box of eggs for the Yellow local breed was not significantly different compared with the Hesa 2 breed and was lower compared with the Super 1 breed. As for fresh cocoon weight in the Yellow local strain, it was significantly lower than in the two controls. Silk shell weight and percentage were also considerably lower in the Yellow local breed than in the Super 1 and Hesa 2 breeds.

It can be concluded that the cocoon weight and silk shell weight and percentage values are lower in the old Yellow local strain than in the white-cocoon breeds used as controls.

It is evident from Table 6 that the Yellow local silkworm strain achieved considerably lower silk filament length and weight, reelability, and raw silk percentage as compared with the two white-cocoon breeds used as controls.

It can be concluded that the Yellow local strain displays high hatchability and survivability at optimal rearing conditions, shorter fifth-instar duration, and a comparatively good reproduction capacity. On the other hand, compared with the two Bulgarian white-cocoon breeds used as controls, it achieves lower values for main productive characteristics such as cocoon weight, silk shell weight and percentage, silk filament length and weight, reelability, and raw silk percentage. Therefore, in order for this breed to be commercialized today, it needs to be improved by way of genetics and breeding.

Silkworm breed improvement is achieved mainly by crossing with breeds that have the desired characteristics and/or manifest high heterosis expression [53,54,55,56]. According to a number of scientists [57,58,59,60,61,62], industrial crossbreeding between two or more initial forms (breeds or lines) with different geographical and genetic origins is one of the most effective methods for creating high-heterosis silkworm hybrids and for their use in production. According to these authors, the superiority of F_1_ hybrids over the average parental values for the main productive traits is in the range of 10–30%. In practice, the most effective method of hybrid creation is the crossbreeding of inter-strain populations of different geographic and genetic origins, and also that of univoltine and bivoltine with polyvoltine breeds [63,64,65].

In order to improve the main quantitative traits of some old breeds with colored cocoons, it is necessary to cross them with highly productive white-cocoon breeds and subsequently select them in order to consolidate valuable economic traits in the offspring while preserving the qualitative traits typical for the old breeds, such as larval markings, cocoon color, and cocoon shape. In this regard, it is of particular importance to know and study the inheritance of these qualitative traits for the hybridization between breeds with colored and white cocoons. According to [66,67,68], the most common marking of larvae is “lack of color”, and it is most often considered the main mutated allele; as a result, other types have appeared. Larvae with the genotype p/p never have any markings, while larvae with the genotype +p/+p have the following three patterns: an “eye spot” (mask) on the dorsal thoracic part and a “half moon” and “star-shaped spots”, located on the dorsal part of the second and fifth abdominal segments, respectively. The +p allele comes in four different varieties, p1, p2, p3, and p4, which determine, in ascending order, the intensity of the larval markings. All of these groups have multiple alleles located on the second chromosome, locus 0.0, and they always determine the traits of the larval markings. Ref [69] has identified the ‘mamo’ gene as a novel regulator of pigmentation in the silkworm *Bombyx mori*, a function that had not previously been suspected based on extensive work in Drosophila. The evidence supporting the role of Bm-mamo in pigmentation is compelling, including high-resolution linkage mapping of two mutant strains, expression profiling, and the reproduction of mutant phenotypes with state-of-the-art RNAi and CRISPR assays.

The cocoon color of Bombyx mandarina, known as the wild silkworm, is homogeneously brownish-yellow. Cocoons of the domesticated silkworm *Bombyx mori* L. are impressively diverse in terms of color and can be divided into three categories according to the type and content of pigments: I—yellow–red cocoons, colored by carotenoids, which can also be golden, pink, and rusty, etc.; II—green cocoons, colored by flavonoids, pale green to deep green in color; and III—white cocoons with traces or no pigment [70,71,72,73]. The absorption and modification of pigments from mulberry leaves in B. mori L. is the key to the formation of colored cocoons. This process is influenced by the genotype of the silkworm and the content of carotenoids in the midgut, hemolymph, and silk gland [74]. These factors influence the determination of the color of the cocoons [72]. In hybridization between the Cambodia breed, which has a yellow–green cocoon, and the white-cocoon breed Japanese 106, Murakami and Ohtsuki [75] found that in F1, all cocoons had a yellow–green color. According to Doira [76] and Lim [67], the genes responsible for yellow cocoon color are known and are as follows: C—golden-yellow; Cd—light yellow; Ci—yellow inner layer of the silk shell; dy—tangerine–yellow hemolymph; Yc—yellow inner layer; Yf—yellow fluorescent; and Yr—yellow–brown. According to [68,77], genes I and Is, known as yellow inhibitors, suppress the action of the Y gene and result in a white cocoon. Kovalev and Sheveleva [78] reported that larvae with yellow hemolymphs are capable of spinning both yellow and white cocoons, while larvae with colorless hemolymphs spin only white cocoons. The Y gene (yellow hemolymph) controls carotenoid uptake in the intestinal mucosa and silk gland. Mutant larvae homozygous for the recessive Y allele inadequately absorb carotenoids from mulberry leaves, resulting in colorless hemolymphs and white cocoons. At least six genes that control carotenoid-based cocoon color function only when co-existing with the dominant Y allele, suggesting that the Y gene plays a central role in cocoon color [71]. In *Bombyx mori* L., the mutation of the carotenoid-binding protein (CBP) (Y locus) and/or absence of the cameo2 gene (C locus) has been shown to result in white-cocoon formation [71,72,77]. According to Lea [66], the genes responsible for green cocoon color are Ga, Gb, and Gc. Ref [79] crossed female moths of the Thai polyvoltine breed with males of the wild silkworm Bombyx mandarina and found that in the first generation, the cocoon color was light green, like that of the wild silkworm. Ref [80] conducted a study with five local silkworm breeds in Iran, including Baghdad, Khorasan Orange, Guilan Orange, and Khorasan, crossed with two white-cocoon breeds, 107 and 110, and found that the colored-cocoon trait dominated over the white-cocoon trait in F1. The breakdown obtained in F2 was three-parts colored and one-part white cocoons. In the first backcross (BC1), the breakdown was one-part colored and one-part white cocoons. The intensity of the cocoon color in F2 and BC1 had significant differences; therefore, it is assumed that the genes responsible for cocoon color in these breeds are represented as multi-alleles in Iranian local breeds of silkworms with colored cocoons. Two main types of pigments are responsible for the cocoon color, namely, ether-soluble yellowish carotenoids and water-soluble green flavonoids [81,82]. Ref [83] analyzed metabolic changes in pigments in the silkworm. The results showed that flavonoids accumulated in the midgut of fifth-instar larvae, and carotenoids accumulated in the silk gland. Cocoon color was found to be closely related to the content of both types of pigments in the silk gland. The pigments are absorbed from mulberry leaves in the midgut and then transported through the hemolymph to the silk gland to produce a cumulative effect. In the silkworm Antheraea yamamai, which spins cocoons with a green–yellow color, Ref [84] determined that some cocoons have a less-yellowish color, called emerald green (EG). The authors studied the inheritance of the EG mutation by crossing individuals that produced EG cocoons with those that produced normally colored cocoons (NG). It was found that F1 offspring from the reciprocal cross between EG and NG individuals produced predominantly NG cocoons. It was concluded that the EG mutation is controlled by a single recessive gene locus.

Lin Zhua and Yu-Qing Zhang [85] conducted studies on the larval tissues and colored cocoons of silkworms of the species *Bombyx mori* L. (Lepidoptera: Bombycidae), which were fed with leaves of a cultivated mulberry, cultivar Husang 32. It was found that mulberry leaves mainly contained four types of pigments: lutein (30.86%), β-carotene (26.3%), chlorophyll a (24.62%), and chlorophyll b (18.21%). The silk glands, hemolymphs, and cocoon shells of six yellow-cocoon breeds were examined. The results showed that, generally, there were two types of carotenoids (lutein and β-carotene) in the silk gland and cocoon shell; little violaxanthin was found in the silk gland, and the pigment found in the hemolymph was mainly lutein.

The shape of the cocoon depends on the movements of the larva during spinning, which in turn is under neurophysiological control [86]. Murakami and Ohtsuki [75] have found that a spindle-shaped cocoon is usually associated with a wavy structure of the silk shell. At the same time, a spindle-shaped cocoon is undesirable from the point of view of silk reeling because it leads to lower reelability [78]. According to [87,88,89], an even cocoon shape contributes to the production of silk threads with a uniform thickness. According to Lea [66], it is difficult to determine a specific gene for a specific cocoon shape because its variation is like a quantitative trait, meaning that it is probably determined by many genes. Kovalev and Sheveleva [78] found that when crossing white-cocoon monobivoltine breeds of the Japanese and Chinese types, the cocoon shape in F1 was always inherited intermediately as a quantitative trait, and in F2, a breakdown with many transitional forms was observed, from an elongated shape with an intercept to an oval shape. When crossing the bivoltine breed C 108, characterized by an oval cocoon shape, with the Cambodia breed, which has a spindle-shaped cocoon, Murakami and Ohtsuki [75] found that in F1, all cocoons were of the shape of C 108, namely oval, while in F2, three-parts of cocoons had an oval shape and one-part was spindle-shaped. According to [90], less variation in the shape of the cocoon was found in F1 hybrids between polyvoltine and bivoltine breeds with oval cocoons compared to F1 hybrids between polyvoltine and bivoltine breeds with elongated intercepted cocoon shapes. In this study, bivoltine hybrids showed less variation in cocoon shape compared to hybrids between polyvoltine and bivoltine breeds. In bivoltine hybrids, crosses between a mother with an oval cocoon shape and a father with an elongated intercepted cocoon shape showed less variation compared to crosses between a mother with an elongated intercepted cocoon shape and a father with an oval cocoon shape.

## 5. Possible Research Work on Improving Old European Silkworm Breeds

As mentioned above, in Bulgaria, the Yellow local strain was the object of purposive selection until the 1960s, after which, it was maintained as genetic material only. As a result, its quantitative characteristics were kept close to the original ones, but without any further improvement. In the first half of the 20th century, the Yellow local strain was sufficiently viable and productive. However, later on, new commercial breeds and F1 hybrids were created, characterized by higher silk shell weights and percentages.

Considering the results obtained in our studies of the Yellow local strain, we came to the conclusion that in order for this breed to be commercialized today, it needs to be improved by way of genetics and breeding. For this purpose, we developed the breeding plan outlined below.

- **Testing the performance of the Yellow local silkworm strain under unfavorable rearing conditions during the fourth and fifth instars.** (Table 7)

This study will aim to assess the sturdiness of the Yellow local strain in order to decide whether the strain also needs viability improvement.

- Study on the inheritance of qualitative and quantitative characteristics in hybridization between the Yellow local strain and the white-cocoon breeds VB1 and HB2 under standard and adverse rearing conditions.

This study will aim to develop a breeding methodology for improving the Yellow local strain by crossing it with white-cocoon breeds. At the same time, it will aim to retain the strain’s typical qualitative characteristics, such as larvae with zebra markings and a yellow cocoon color.

The VB1 breed is of the Japanese type; the larvae have normal markings and spin white cocoons with an elongated shape and slight constriction. The HB2 breed is of the Chinese type, characterized by plain larvae and white oval cocoons. Both breeds possess comparatively high tolerances to rearing at adverse conditions and moderate productivity. The following generations will be studied: P1, P2, F1, F2, F3, and BC.

- **Study on F1 crosses between the Yellow local strain and some highly productive white-cocoon breeds.**

This study will aim to test the F1 hybrids in order to use the Yellow local breed as one of the parents of F1 hybrids demonstrating higher productivity while retaining zebra larval markings and yellow cocoon color. The following two F1 hybrids will be tested: Super 1 × Yellow local and Hesa2 × Yellow local. Depending on the results obtained, some F2, F3, and BC generations will also be further produced and investigated, aiming at the possible creation of pure lines with the quantitative characteristics of the Yellow local breed but with higher cocoon and shell weights and raw silk percentages.

## 6. ARACNE Project

The ARACNE project (https://aracneproject.eu/, accessed on 19 September 2025), which started in March 2023, has a duration of 36 months and involves 11 partners and 3 associated partners from seven European Union (EU) and non-EU countries.

The project, whose name is inspired by the weaver transformed into a spider by the goddess Athena in Greek mythology, aims to exploit silk as the common element of pan-European culture and history. ARACNE aims to contribute to the creation of a broad and interconnected silk-related innovation ecosystem in Europe, including the industrial sector, and is intended as a tool for expressing cultural and landscape heritage, thus connecting culture, tradition, and new industrial production within an ideal network of exchanges and visions.

The aim of the activities concerning old European silkworm breeds is to gradually re-introduce local silkworm breeds to individuals and stakeholders who are interested in rearing them either for educational purposes, cocoon production purposes, artistic purposes, or any other purpose that such stakeholders find suitable. By connecting with local silkworm farmers and giving them the opportunity to rear such local silkworm breeds, the project attempts to re-introduce a sense of identity and link modern practices with local traditions.

## 7. Conclusions

It can be concluded that the old silkworm breeds Yellow local and White Baghdad, reared in Bulgaria during the 1920s and 1930s, were generally characterized by non-uniform larval color, larval markings, cocoon color, and cocoon shape, as well as a comparatively high cocoon weight but lower silk shell percentages and filament lengths. In addition, they were comparatively tolerant to NPV disease. Due to their long persistence in Bulgaria, they have become well adapted to the local food and climatic conditions.

At present, the Yellow local strain displays high hatchability and survivability, comparatively shorter fifth-instar duration, and a good reproduction capacity. On the other hand, compared with modern commercial Bulgarian white-cocoon breeds, it manifests lower values of main productive characteristics, such as cocoon weight, silk shell weight and percentage, silk filament length and weight, reelability, and raw silk percentage. Therefore, in order for this breed to be commercialized today, its silk yielding traits must be improved via genetics and breeding while preserving its qualitative characteristics.

## Figures and Tables

**Table 2:** Different types of larval color and markings in the silkworm breeds Yellow local and White Baghdad.

Silkworm Breed, Larval Color, and Markings Groups	Larval Color		Larval Markings			
	White	Gray–Brown	Plain	with Masks	with Moons	Zebra
Yellow local						
1	X		X			
2	X			X	X	
3	X			X	X	
4	X					
5	X			X	X	X
6				X	X	X
7		X		X		X
8		X		X	X	X
White Baghdad						
1	X		X			
2	X			X	X	
3	X			X	X	
4	X			X	X	
5	X			X	X	
6	X			X		X
7	X			X	X	X
8	X			X	X	X
9	X			X	X	X
10		X		X	X	
11		X		X	X	
12		X		X	X	
13		X		X	X	
14		X		X	X	X
15		X		X	X	X

**Table 3:** Cocoon size, renditta *, and silk filament length of yellow-cocoon breeds **.

№	Breed	Cocoon Size				Renditta, kg		Silk Filament Length, m	
		Grade I		Grade II					
		Length, cm	Width, cm	Length, cm	Width, cm	Grade I	Grade II	Grade I	Grade II
1	Yellow Italian	3.61	1.69	3.51	1.71	3.251	3.439	713.6	583
2	Yellow local	3.55	1.71	3.57	1.69	3.192	3.609	657	666.5

* ”renditta” means the quantity of cocoons necessary to produce 1 kg of raw silk. ** no standard error or other measures of dispersion were reported in the original reference.

**Table 4:** Raw silk titer, elasticity, and strength of yellow-cocoon breeds.

№	Breed	Titer, Denier						Elasticity						Strength					
		Grade I			Grade II			Grade I			Grade II			Grade I			Grade II		
		Min.	Max.	Aver.	Min.	Max.	Aver.	Min.	Max.	Aver.	Min.	Max.	Aver.	Min.	Max.	Aver.	Min.	Max.	Average
1	Yellow Italian	11	16.6	13.49	10.4	16.4	13.18	63	240	171.7	38	258	147.5	32	62	47.8	26	53	40.7
2	Yellow local	10	15	12.47	8	15.4	12.25	64	242	167.55	34	235	139.05	30	55	43.9	25	52	40.07

**Table 5:** Breeding characteristics in the Yellow local strain: averages for 2023 and 2024.

Breed	Hatchability (%)	Larval Period Duration, h	5th-Instar Duration, h	Cocoon Yield by One Box of Silkworm Eggs, kg	Pupation Rate, %	Fresh Cocoon Weight, mg	Silk Shell Weight, mg	Silk Shell Percentage %
**Yellow local**	98.09	698	147 ***	29.69	90.92 *	1782 **	318 ***	17.85 ***
**Super 1**	98.06	718 *	166	33.20 ***	92.10 ***	1954 ***	407	20.83 **
**Hesa 2 (control)**	97.13	700	170	30.37	89.80	1842	395	21.44

* *p* < 0.05; ** *p* < 0.01; and *** *p* < 0.001.

**Table 6:** Silk filament technological characteristics in the Yellow local strain: averages for 2023 and 2024.

Breed	Filament Length, m	Silk Filament Weight, mg	Reelabilty, %	Raw Silk Percentage, %
**Yellow local**	925 ***	272 ***	87.78 ***	36.33 ***
**Super 1**	1168 *	354	90.05	38.67
**Hesa 2 (control)**	1225	350	90.53	39.65

* *p* < 0.05; ** *p* < 0.01; and *** *p* < 0.001.

**Table 7:** The adverse 4th- and 5th-instar silkworm larvae rearing conditions.

Mode of Silkworm Rearing	Temperature, °C	Relative Air Humidity, %	Feeding Space	Feeding Amount	Ventilation
**Adverse rearing conditions**	29–30	80–90%	11 M^2^ for 1 box of silkworms	1 feeding daily	Closed windows and door
**Standard technology**	23–25	55–70%	22 M^2^ for 1 box of silkworms	2 feedings daily	Two windows open; door open if necessary

## Data Availability

No new data were created or analyzed in this study. Data sharing is not applicable to this article.

## References

[1] Bajwa G.A., Ahmed S., Shah M., Adnan M. (2017). Genetic diversity analysis of mulberry silkworm (*Bombyx mori* L.) strains using RAPD markers. J. Anim. Plant Sci..

[2] Bindroo B., Moorthy S.M. (2014). Genetic Divergence, Implication of Diversity, and Conservation of Silkworm, Bombyx mori. Int. J. Biodivers..

[3] Chandrakanth N., Moorthy S.M., Anusha P., Dayananda, Ashwath S.K., Kumar V. (2014). Evaluation of genetic diversity in silkworm (*Bombyx mori* L.) strains using microsatellite markers. Int. J. Biotechnol. Allied Fields.

[4] Chikkaswamy B.K., Paramanik R.C. (2015). Study on Genetic diversity and relationship of silkworm varieties using RAPD molecular marker. Int. J. Curr. Res. Acad. Rev..

[5] Devi K.I., Ponnuvel K.M., Singh L.S., Singh K.C., Dutta K. (2012). Genetic Diversity among Indian Oak Tasar Silkworms, Anthereae proyeli J. Revealed by ISSR markers. Int. J. Ind. Entomol..

[6] Jingade A.H., Vijayan K., Somasundaram P., Srivasababu G.K., Kamble C.K. (2011). A Review of the Implications of Heterozygosity and Inbreeding on Germplasm Biodiversity and Its Conservation in the Silkworm, Bombyx mori L. J. Insect Sci..

[7] Kumari A., Tripathi N.K., Bano R. (2015). Agglomerative hierarchical clustering to study the diversity in *Bombyx mori* L. races on the basis of weight gain characteristics. Int. J. Recent Sci. Res..

[8] Moorthy S.M., Chandrakanth N., Ashwath S.K., Rao V.K., Bindroo B.B. (2013). Genetic diversity analysis using RAPD marker in some silkworm breeds of *Bombyx mori* L.. Ann. Biol. Resour..

[9] Nezhad M.S., Mirhosseini S.Z., Garahveysi S., Mavvapour M., Seidavi A.R., Naserani M. (2010). Genetic diversity and classification of 51 strains of silkworm *Bombyx mori* L. (Lepidoptera: Bombycidae) germplasm based on larval phenotypic data using Ward’s and UPGMA methods. Afr. J. Biotechnol..

[10] Seong W.K., Min J.K., Seong R.K., Jeong S.P., Kee Y.K., Ki H.K., Woori K., Iksoo K. (2022). Whole-genome sequences of 37breeding line *Bombyx mori* strains and their phenotypes established since 1960s. Sci. Data.

[11] Dushev T. (1929). Otchet na Bubarskata Opitna i Kontrolna Stancia vav Vratsa za 1927 Godina (Report of the Sericulture Experiment and Control Station in Vratsa for Year 1927).

[12] Dushev T. (1933). Otchet na Bubarskata Opitna i Kontrolna Stancia vav Vratsa za 1928 i 1929 godina (Report of the Sericulture Experiment and Control Station in Vratsa for the Years 1928 and 1929).

[13] Dushev T. (1933). Otchet na Bubarskata Opitna i Kontrolna Stancia vav Vratsa za 1930 Godina (Report of the Sericulture Experiment and Control Station in Vratsa for the year 1930).

[14] Dushev T. (1935). Bubarstvoto—Negovoto Znachenie za Narodnoto Stopanstvo i Roljata mu Dnes pri Zadovoljavane Nujdite na Stranata ot Tekstilni Vlakna (The Sericulture—Its Importance for the National Economy and its Role Today in Satisfying the Country’s Needs of Textile Fibers). Bubarski Pregled.

[15] Dushev T. (1935). Bubarstvoto vav Vrachanska Oblast i Merki za Podobrjavaneto mu (The Sericulture in Vratsa’s Region and Measures for its Improvement). Bubarski Pregled.

[16] Dushev T. (1935). Opiti i Prouchvania na Bubarskata Opitna i Kontrolna Stancia vav Vratsa prez Perioda 1931–1932 Godina (Experiments and Studies of the Sericulture Experiment and Control Station in Vratsa During 1931–1932).

[17] Dushev T. (1929). Bubarstvo (Sericulture).

[18] Dushev T. (1930). Chernicata v Balgaria (The Mulberry in Bulgaria). Balgarska koprina.

[19] Dushev T. (1931). Prinos kam Prouchvane na Bjalata Bagdatska rasa, Otglejdana v Balgaria (Contribution to the Study of White Baghdad Strain, Reared in Bulgaria).

[20] Dushev T. (1933). Otchet na Darjavnata Bubarska Opitna i Kontrolna Stancija v grad Vratsa (Annual Report of the State Sericultural and Control Station in Vratsa).

[21] Dushev T. (1935). Opiti i Prouchvanija na Darjavnata Bubarska Opitna i Kontrolna Stancija v grad Vratsa prez Perioda 1933–1934 (Experiments and Studies of the State Sericultural and Control Station in Vratsa During the Period 1933–1934).

[22] Dushev T. (1937). Njakoi Prouchvania v Oblastta na Chernicharstvoto (Some Research in the Field of Moriculture).

[23] Dushev T. (1941). Savremenno Bubarstvo i Chernicharstvo (Modern Sericulture and Moriculture).

[24] Botev S., Kovachev J. (1930). Zemedelieto v Balgaria (The agriculture in Bulgaria).

[25] Georgiev J. (1903). Grad Vratsa (The Town of Vratsa).

[26] Irechek K. (1883). Stari Pateshestvia po Balgaria (Old Travels in Bulgaria). Spis. Balgarskoto Knijovno Drujestvo.

[27] Jolov A., Petkov M., Penkov I. (1983). Kratak Ocherk za Razvitieto na Bubarstvoto v Balgaria do 9 Septemvri 1944 Godina (Short outline of the Sericulture Development in Bulgaria Until 9th September 1944). Izv. Nac. Selskostop. Muzei.

[28] Kanic F. (1932). Dunavska Balgaria i Balkana (Danubian Bulgaria and the Balkan).

[29] Kozukharov I. (1970). Bubarstvoto v Balgaria (The Sericulture in Bulgaria).

[30] Delev S. (1907). Razvoj na Koprinarstvoto v Balgaria i Negovoto Badeshte (Silk Industry Development in Bulgaria and Its Future).

[31] Vasileva N. (1985). Bubarstvoto i Koprinarstvoto kato Narodni Zanajati vav Velikotarnovskia kraj (The Sericulture and silk Textile as People’s Crafts in Veliko Tarnovo Region). Ethnogr. Mus. Bull..

[32] Dimitrova N. (2002). Tradicionno bubarstvo i koprinarstvo vav Vrachansko, Severozapadna Balgaria—obshnosti, tradicii, nalichnost (Traditional Sericulture and Silk Industry in Vratsa’s Region, North—West Bulgaria—Communities, Traditions, Availability. Ethnogr. Mus. Bull..

[33] Dojnov N. (1975). Bubarstvo–proizvodstvo na koprina i koprineni takani vav Vratsa–tradicia i inovacii (Sericulture–silk and silk fabrics production in Vratsa–tradition and innovations). Vestnik Otechestven zov.

[34] Jordanov H. (1896). Sastojanie na Bubarstvoto prez 1885 Godina (the Sericulture Status in 1885).

[35] Jocov D. (1932). Kulturno-Istoricheska Istoria na Vratsa (Cultural—Historical History of Vratsa).

[36] Angelov I. (1966). Vratsa—gradat na staroto bubarstvo i koprinarstvo (Vratsa—the city of old sericulture and silk textile). Vestnik Otechestven zov.

[37] Ivanchev M. (1928). Proizvodstvo na Pashkuli ot Koprinenata buba i Targovija s tjah (Production of Cocoons from the Silkworm and the Trade with them).

[38] Dimitrova N. (2003). Narodni Znanija za Koprinenata buba i Otglejdaneto i pri Domashni Uslovia vav Vrachansko (po Materiali ot Kraja na 19 i Nachaloto na 20 vek). (People’s Knowledge About the Silkworm and its Rearing at Home Conditions in Vratsa’s Region (by Materials from the end of 19th and the Beginning of 20th Century). Rastitelnijat i Jivotinski Svjat v Tradicionnata Kultura na Balgarite. Ethnogr. Mus. Bull..

[39] Kosev K. (1968). Za kapitalisticheskoto Razvitie na Balgarskite zemi prez 60–70te godini na 19 vek (About the Capitalist Development of Bulgarian Lands During 60–70th Years of 19th Century).

[40] Kozukharov I. (1956). Istoria, Zadachi i Postijenia na Bubarskata Opitna i Kontrolna Stancia v grad Vratsa 1906–1956 Godina (History, Tasks and Achievements of the Sericulture Experiment and Control Station in the City of Vratsa 1906–1956).

[41] (1933). Otchet na Balgarskata Zemedelska Banka ot 1926 do 1932 Godina (Report of the Bulgarian Agricultural Bank for the Period from 1926 to 1932).

[42] (1945). Otchet na Balgarskata Zemedelska Banka ot 1934 do 1945 Godina (Report of the Bulgarian Agricultural Bank for the Period from 1926 to 1945).

[43] (1942). Otcheti, Protokoli, Dokladi i Reshenia na 3 i 4 Redovni Obshti Sabrania na Zemedelskata Kamara (Reports, Protocols and Decisions of the 3rd and 4th Current join Assemblies of the Agricultural Chamber).

[44] Jolov A., Penkov I., Petkov M. (1978). Bubarstvo—Nauchno-Technicheska Koncepcia i Prognoza za Perioda 1980–2000 (Sericulture—Scientific—Technical Conception and Prognosis for the Period 1980–2000).

[45] Kozukharov I. (1966). 50 Godini OSB Vratsa (1906–1956 Godina), (50 Years SES Vratsa (1906–1956)).

[46] Jolov A., Petkov N., Penkov I. (1987). Za nov Podem na Bubarstvoto vav Vrachanski Okrag (for a New Raise of the Sericulture in Vratsa’s District).

[47] (1944). Otchet na Okoliiskoto Agronomstvo v Bjala Slatina (Report of the Regional Agronomic Office in the Town of Bjala Slatina).

[48] Tzenov P. (2023). Annual Scientific Report.

[49] Tzenov P. (2024). Annual Scientific Report.

[50] (1942). Otchet na Okoliiskoto Agronomstvo v Oriahovo (Report of the Regional Agronomic office in the Town of Oriahovo).

[51] Gecheva T. (1948). Otchet na Bubarskata Opitna i Kontrolna Stancia (Report of the Sericulture Experiment and Control Station).

[52] (1947). Dokladi i Instrukcii za Zadachite i Metodite na Rabota pri Bubarskite Opitni i Kontrolni Instituti v Balgaria (Reports and Instructions for the Work of the Sericulture Experimental and Control Institutes in Bulgaria).

[53] Gupta B., Verma M., Singh K. (1992). Promising bi x bi hybrids of silkworm, *Bombyx mori* L.. Sericologia.

[54] Kang P.D., Kim K.M. (1998). Breeding of “Chungangjam”, a high silk yelding new silkworm variety for spring rearing season. RDA—J. Ind. Crop Sci..

[55] Kang P.D., Kim K.M., Sohn B.H., Lee S.O., Woo S.O., Hong S.G. (2001). Breeding of new silkworm variety, Chunsujam, with a high silk yielding for spring rearing season. Int. J. Indust. Entomol..

[56] Kantaratanakul S., Tharvarnanukulkit C., Wongthang S., Shreonyng S. (1987). Heterosis in F1 hybrid between polyvoltine and bivoltine silkworm, *Bombyx mori* L.. Sericologia.

[57] Hyrata Y. (1985). Economical characters in the double crosses of the silkworm Bombyx mori L. Acta. Sericologica.

[58] Lakshmi H., Babu M.R., Prasad J., Chandrashekharaiah C. (2008). Identification of promising cross breed APM_1_ × APS_98_ in mulberry silkworm through manifestation of hybrid vigour. Bull. Ind. Acad. Seri..

[59] Lea H.Z. (1998). Breeding of bivoltine silkworm hybrids DPO308 and DPO314 in Sri Lanka: A simplified and practical approach. Korean J. Seric. Sci..

[60] Narayanaswamy K.C., Manjunath G., Reddy D.N. (2007). Assessment of performance of three—way crosses involving Pure Mysore as female and CSR hybrids as male parents. Bull. Ind. Acad. Seri..

[61] Rao D., Singh R., Premalatha V., Sudha V.N., Kariappa B.K., Dandin S.B. (2004). Studies on Boil-off Loss Ratio in the Cocoon Shells of Multivoltine x Bivoltine Hybrids of Silkworm, Bombyx mori L. J. Indust. Entomol..

[62] Sannappa B., Devaiah M.C., Govindan R., Ramakrishna N. (2002). Effect of feeding regimes on cocoon traits of *Bombyx mori* L.. Insect Environ..

[63] Rao C., Chandrashekariah G.P., Rmesh C., Basha K., Seshagiri S.V., Nagaraju H. (2004). Evaluation of polyvoltine hybrids based on silk productivity in silkworm, *Bombyx mori*. Int. J. Indust. Entomol..

[64] Sofi A.M., Malik G.N., Malik M.A., Malik F. (2005). Estimation of heterosis and gene action among newly evolved breeds of bivoltine silkworm *Bombyx mori* L.. Bull. Ind. Acad. Seri..

[65] Sudhakara R.P., Narasimha A.R., Mamatha M., Sowmyashree T.S., Bashir I., Ilahi I. (2007). Development of new robust bivoltine silkworm hybrid SR2 x SR5 for rearing throughout the year. Int. J. Indust. Entomol..

[66] Lea H.Z. (1993). Principles and Techniques of Silkworm Breeding.

[67] Lim S.H. (1995). Principles and Practices in Sericulture.

[68] Tazima P. (1964). The Genetics of the Silkworm.

[69] Wu S., Tong X., Peng C., Luo J., Zhang C., Lu K., Li C., Ding X., Duan X., Lu Y. (2024). The BTB-ZF gene Bm-mamo regulates pigmentation in silkworm caterpillars. Genet. Genom..

[70] Daimon T., Hirayama C., Kanai M., Ruike Y., Meng Y., Kosegawa E., Nakamura M., Tsujimoto G., Katsuma S., Shimada T. (2010). The silkworm Green b locus encodes a quercetin 5-O-glucosyltransferase that produces green cocoons with UV-shielding properties. Proc. Natl. Acad. Sci. USA.

[71] Sakudoh T., Sezutsu H., Nakashima T., Kobayashi I., Fujimoto H., Uchino K., Banno Y., Iwano H., Tamura T., Kataoka H. (2007). Carotenoid silk coloration is controlled by a carotenoid-binding protein, a product of the Yellow blood gene. Proc. Natl. Acad. Sci. USA.

[72] Sakudoh T., Iizuka T., Narukawa J., Sezutsu H., Kobayashi I., Kuwazaki S., Banno Y., Kitamura A., Sugiyama H., Takada N. (2010). A CD36-related transmembrane protein is coordinated with an intracellular lipid26 binding protein in selective carotenoid transport for cocoon coloration. J. Biol. Chem..

[73] Sakudoh T., Kuwazaki S., Iizuka T., Narukawa J., Yamamoto K., Uchino K., Sezutsu H., Banno Y., Tsuchida K. (2013). CD36 homolog divergence is responsible for the selectivity of carotenoid species migration to the silk gland of the silkworm *Bombyx mori*. J. Lipid Res..

[74] Yaru L., Jiangwen L., Erxia A., Bo L., Yinqiu W., Xiang C., Kunpeng L., Shubo L., Hai H., Minjin H. Deciphering the Genetic Basis of Silkworm Cocoon Colors Provides New Insights into Biological 2 Coloration and Phenotypic Diversification. https://academic.oup.com/mbe/advance-article/doi/10.1093/molbev/msad017/7013732.

[75] Murakami A., Ohtsuki Y. (1989). Genetic studies on tropical races of silkworm *Bombyx Mori* L. with special reference to crossbreeding strategy between tropical and temperate race. I. Genetic nature of the tropical multivoltine strain Cambodge. Jpn. Agric. Res. Q..

[76] Doira H. (1992). Genetical Stocks and Mutations of Bombyx mori: Important Genetic Resources. Linkage Maps and List of Genetical Stocks Maintained in Kyushu University.

[77] Banno Y., Fujii H., Kawaguchi Y., Yamamoto K., Nishikawa H., Nishizaka A., Tamura K., Eguchi S. (2005). A Guide to the Silkworm Mutants Gene Name and Gene Symbol—Silkworm Resource Section.

[78] Kovalev P.A., Sheveleva A.A. (1966). Silkworm Breeding and Egg Production.

[79] Wilai S., Yokoyama T., Ninagi O. (2015). Some quantitative characters from the crossing of Thai multivoltine silkworm, *Bombyx mori* L. and mulberry wild silkworm, Bombyx mandarina Moore. Int. J. Wild Silkmoth Silk.

[80] Mirhosseini S.Z., Nematollahian S.H., Vishkaee S., Bizhannia A.R., Seidavi A.R., Mavvajpour M., Ghanipoor M. (2010). Performance and Cocoon Color Segregation Manifested in Hybrids Between Native Races and Two Commercial Lines of Silkworm in Five Successive Generations. Acad. J. Entomol..

[81] Bhosale P., Bernstein P.S. (2007). Vertebrate and invertebrate carotenoid-binding proteins. Arch. Biochem. Biophys..

[82] Tsuchida K., Katagiri C., Tanaka Y., Tabunoki H., Sato R., Maekawa H., Takada N., Banno Y., Fujii H., Wells M.A. (2004). The basis for colorless hemolymph and cocoons in the Y-gene recessive *Bombyx mori* mutants: A defect in the cellular uptake of carotenoids. J. Insect Physiol..

[83] Xu Y., Wang Y., Lin J., Zhong Y., Lin B. (2010). Metabolic variations of the main pigment substances in silkworm larvae spinning colored cocoons. Sci. Seric..

[84] Yasuhiro Y., Shin-ichiro A., Hisanori B., Ken S. (2017). Inheritance of cocoon color mutation and heterosis in Antheraea yamamai. Int. J. Wild Silkmoth Silk.

[85] Lin Z., Yu-Qing Z. (2014). Identification and analysis of the pigment composition and sources in the colored cocoon of the silkworm, *Bombyx mori*, by HPLC-DAD. J. Insect Sci..

[86] Murakami A. (1989). Genetic studies on tropical races of silkworm *Bombyx Mori* L. with special reference to cross breeding strategy between tropical and temperature races II. Multivoltine silkworm strains in Japan and their origin. J. Assoc. Res. Otolaryngol..

[87] Mano Y. (1994). Comprehensive Report on Silkworm Breeding.

[88] Ravindra S., Kalpana G.V., Sudhakara Rao P., Ahsan M.M. (1998). Studies on cocoon shapes in different crosses of the mulberry silkworm, *Bombyx mori* L.. Indian J. Seric..

[89] Ravindra S., Raghavendra Rao D., Premalatha V., Monda S., Kariappa B.K., Jayaswal K.P., Datta R.K. (2001). Manifestation of hybrid vigour and cocoon shape variability in F1 hybrids of the mulberry silkworm, *Bombyx mori* L.. Int. J. Indust. Entomol..

[90] Ramesh Babu H., Lakshmi J., Seetha R., Chandrasekharaiah C., Raju P.J. (2010). Studies on cocoon shape variability in different crosses of silkworm Bombyx mori L.. J. Exp. Zool..

